# Neurological Outcomes of Newborn Screening‐Identified Isovaleric Acidemia: A Case Series Exploring Initial C5 Acylcarnitine Levels

**DOI:** 10.1002/jmd2.70094

**Published:** 2026-05-18

**Authors:** Sharmila Kiss, Gary Lazarovski, Maureen Evans

**Affiliations:** ^1^ Department of Metabolic Medicine The Royal Children's Hospital Parkville Victoria Australia; ^2^ Victorian Clinical Genetics Services, Murdoch Children's Research Institute Parkville Victoria Australia

## Abstract

Isovaleric acidemia (IVA) is a rare autosomal recessive disorder caused by isovaleryl‐CoA dehydrogenase deficiency, leading to toxic metabolite accumulation and potentially life‐threatening metabolic crises. Newborn screening (NBS) has enabled early detection through elevated C5 acylcarnitine levels, yet the prognostic value of initial C5 concentrations remains unclear. This single‐center retrospective study examined 10 Australian patients diagnosed with IVA via NBS between 2004 and 2025. Patients were stratified as “mild” or “classic” based on initial C5 levels and clinical severity. Developmental outcomes were assessed using standardized tools and clinical evaluations. Despite biochemical evidence of metabolic instability, including hyperammonemia, acidosis, and hospitalizations, no patients demonstrated neurological impairment on follow‐up. Notably, individuals with markedly elevated C5 levels (up to 63.6 μmol/L) remained neurologically intact, suggesting that early diagnosis and timely metabolic management (protein restriction, carnitine/glycine supplementation, emergency protocols) may mitigate long‐term CNS involvement. Dietary practices varied, with some patients maintaining protein restriction due to self‐limited intake. Our findings reveal substantial heterogeneity in biochemical profiles and clinical trajectories, with minimal correlation between initial C5 levels and neurodevelopmental outcomes. These results align with prior studies questioning the predictive value of isolated NBS markers and support a more nuanced, individualized approach to IVA management. Limitations include small sample size, retrospective design, and incomplete standardized neurocognitive testing. Further prospective studies incorporating genotype data and formal assessments are needed to refine risk stratification and optimize long‐term care strategies.

## Key Points

- *Dissociation between C5 levels and prognosis*. Initial newborn screening C5 acylcarnitine concentrations show minimal correlation with long‐term neurodevelopmental outcomes, as even patients with marked elevations (up to 63.6 umol/L) remained neurologically intact.
- *Efficacy of early intervention*. Early diagnosis via NBS, followed by prompt implementation of protein restriction, carnitine/glycine supplementation, and rigorous emergency protocols, appears highly effective in preventing permanent central nervous system (CNS) involvement in both “mild” and “classic” IVA phenotypes.
- *Clinical heterogeneity and management*. The study highlights significant biochemical and clinical heterogeneity in IVA, suggesting that while initial C5 levels are essential for detection, they are insufficient for risk stratification, necessitating a more individualized approach to long‐term metabolic management.

## 1 Introduction

IVA is an autosomal recessive disorder caused by a deficiency of isovaleryl‐CoA dehydrogenase (IVD), a key enzyme in leucine catabolism. This defect leads to the accumulation of toxic metabolites such as isovaleric acid, resulting in potentially life‐threatening metabolic disturbances [1].

Clinically, IVA presents with a wide phenotypic spectrum, ranging from acute neonatal crises to chronic intermittent forms and even asymptomatic individuals identified through newborn screening (NBS) [2, 3]. The neonatal form typically manifests within the first days of life, often triggered by catabolic stress that may be associated with feeding establishment or infection. Symptoms may include vomiting, lethargy, hypotonia, seizures, and rapid progression to coma or death if untreated [4, 5]. Laboratory findings often reveal metabolic acidosis, hyperammonemia, and an elevated anion gap, with a characteristic “sweaty feet” odor due to isovaleric acid accumulation [1, 5]. The chronic intermittent form, presenting later in infancy or childhood, is marked by episodic decompensations during illness or reduced energy intake, with symptoms such as vomiting, encephalopathy, and ataxia. Between episodes, patients may remain clinically well, though repeated crises can lead to cumulative neurological injury [6, 7].

NBS programs have transformed IVA detection, primarily through elevated C5 acylcarnitine (isovalerylcarnitine) levels in dried blood spots [3, 8]. In Australia, IVA is included in the national NBS panel, allowing for early diagnosis and intervention. However, interpreting C5 levels remains challenging, as elevations may reflect both classical and mild variants [6, 7, 9, 10]. Furthermore, the lack of global standardization and harmonization across neonatal screening programs complicates the comparison of results between jurisdictions; a specific C5 concentration reported by one laboratory may not be analytically equivalent to that of another [7, 11]. Emerging evidence suggests that within a consistent screening framework, initial C5 concentrations may correlate with residual enzyme activity and clinical severity, offering a potential biomarker for risk stratification and individualized management [6, 8].

Despite widespread NBS implementation, data on long‐term outcomes of IVA patients identified through screening in Australia remain limited. The relationship between initial C5 levels and disease trajectory has not been systematically explored. Understanding this correlation could inform treatment decisions, including the degree of protein restriction, use of glycine and carnitine supplementation, and frequency of metabolic monitoring.

This study presents a retrospective analysis of 10 Australian patients diagnosed with IVA via NBS, examining initial C5 levels, frequency of metabolic decompensation episodes, dietary modifications, and neurodevelopmental outcomes. By stratifying patients based on biochemical severity, we aim to identify patterns that may guide personalized treatment and improve long‐term prognosis.

## 2 Patients and Methods

### 2.1 Study Population

This was a single‐center retrospective cohort study of all infants identified with an elevation of C5 acylcarnitine in the state of Victoria in Australia NBS program between 2004 and 2025. Following NBS, diagnostic confirmation was achieved via the identification of elevated urinary isovalerylglycine and/or *IVD* gene sequencing. Urinary organic acids were analyzed using gas chromatography–mass spectrometry (GC–MS). Semiquantitative analysis of specific metabolites, including isovalerylglycine, was performed according to the methodology described by Pitt et al. [12]. Of the 35 cases identified during this period, 10 met the inclusion criteria of having longitudinal follow‐up data available at the Metabolic Medicine clinic at The Royal Children's Hospital. Patients were included if they had [1] a confirmed diagnosis of IVA (biochemical and/or DNA sequencing) and [2] longitudinal clinical follow‐up data at our metabolic service. No patients identified during this period were excluded, ensuring a non‐biased representation of the local IVA population.

### 2.2 Classifications and Definitions

Consistent with prior studies, IVA was categorized as either “mild” or “classic.” The mild form was defined by a less severe or potentially benign clinical course, and/or an initial NBS C5 acylcarnitine concentration below 5.6 μmol/L [10]. Classic IVA was characterized by a C5 concentration exceeding 5.6 μmol/L in the first NBS sample, and/or clinical indicators of a more severe phenotype, such as early‐onset (EO) metabolic decompensation.

Metabolic decompensation was considered present if the patient required hospitalization (minimum one overnight stay) due to biochemical instability or exhibited clinical signs of decompensation, including severe metabolic acidosis, hyperammonaemic encephalopathy, or altered consciousness [10]. Episodes involving impaired consciousness and/or intensive care unit admission were classified as severe. EO decompensation was defined as occurring within the neonatal period (< 29 days of life), while later presentations were classified as late‐onset (LO) [10].

Developmental outcomes were assessed using a combination of standardized tools and clinical evaluations. Formal assessments included the Brigance Kinder Developmental Screen [13], the Wechsler Intelligence Scale for Children—Fourth Edition [14], the Wechsler Preschool and Primary Scale of Intelligence—Third Edition [15], and the Vineland Adaptive Behavior Scales—Second Edition [16]. Not all participants underwent standardized testing both before and after intervention. For those without formal assessments, developmental status was determined based on clinical evaluations documented by pediatricians/Metabolic physicians in patient records.

Using available data, each child's developmental profile was categorized into one of four levels: no impairment, mild, moderate, or severe impairment. “No impairment” was assigned when neurological examinations were normal and there were no significant developmental delays. “Mild impairment” indicated persistent neurological abnormalities and/or delays in a single domain, such as motor or language. “Moderate impairment” referred to persistent abnormalities and/or delays in two domains. “Severe impairment” was defined by the presence of spastic quadriplegia or profound developmental delay.

This classification framework reflects a widely accepted approach for evaluating neurodevelopmental outcomes and has been applied in previous studies across various metabolic and genetic conditions [13, 14, 15, 16].

### 2.3 Data Analysis

Patients were stratified into two cohorts (Mild and Classic) based on initial C5 levels. Comparative analysis was performed between these groups regarding the frequency of metabolic decompensations, peak ammonia levels, and maintenance therapy requirements. Descriptive statistics (medians and ranges) were used to identify clinical patterns associated with each phenotype.

## 3 Results

### 3.1 Study Cohort and Clinical Characteristics

This case series included 10 children (3 female, 7 male) with confirmed IVA by biochemical and/or DNA sequencing (Tables 1 and 2).

**TABLE 1:** Patient demographics and clinical trajectory from newborn screening to last follow‐up.

Patient	Sex	Gestational age	Birth weight (g)	Co‐morbidities	Age at NBS sampling (h)	Symptomatic at NBS referral	Symptomatic at time of diagnosis	Age at last follow up (years and months)	Neurodevelopmental outcome
1	M	32	1800	RDS needing CPAP for 24 h	48	No	No	2 years 3 months	Delayed gross motor skills till 20 months
2	M	35+6	2450	Hypoglycaemia treated with feeds	49	Yes (hypoglycaemia, lethargic)	Yes	13 years 5 months	Normal
3	M	39	4000	Nil	48	No	No	12 years 9 months	Normal
4	M	38	3580	Nil	49	No	Yes	3 years 0 months	Normal
5	M	39	3500	Nil	58	Yes (poor feeding, lethargy)	Yes	5 years 0 months	Normal
6	F	40	3030	Nil	52	No	No	15 years 4 months	Normal
7	F	39+3	3480	Nil	50	No	No	7 years 6 months	Normal
8	M	41	3200	Nil	54	No	No	18 years 2 months	Normal
9	M	40	4316	Nil	64	No	No	18 years 2 months	Normal
10	F	39+1	3800	Nil	35	Yes (poor feeding)	Yes	18 years 3 months	Normal

**TABLE 2:** Biochemical phenotype, molecular results (if applicable), clinical stability metrics.

Patient	C5 1st NBS sample (0–0.8 μmol/L)	Urine organic acids	Molecular results	IVA type	Pharmacological management	Illness‐related hospital admissions (number of episodes)	Onset type	Ammonia max (< 50 μmol/L)
1	0.959	Isovaleryl glycine +	VUS heterozygous c.224A>G (p.Asn75Ser)	Mild	None	0	—	N/A
2	17	Isovaleryl glycine > +++	Not done	Classic	Carnitine + Glycine	10	LO	91
3	1.2	Isovaleryl glycine +	Not done	Mild	None	0	—	N/A
4	4.02	Isovaleryl glycine > +++	VUS heterozygous c.862C>G (p.Leu288Val)	Mild	Carnitine + Glycine	4	LO	—
5	9.92	Isovaleryl glycine > +++ 3‐hydroxyisovaleric acid	Not done	Classic	Carnitine + Glycine	9	LO	90
6	3.68	Isovaleryl glycine ++	Not done	Mild	None	4	LO	—
7	2.08	Isovaleryl glycine ++	Not done	Mild	Carnitine + Glycine	5	LO	—
8	4.39	Isovaleryl glycine +	Not done	Mild	Carnitine + Glycine	3	LO	—
9	3.99	Isovaleryl glycine +++	Not done	Mild	Carnitine only	3	LO	—
10	63.6	Isovaleryl glycine +++	Not done	Classic	Carnitine + Glycine	2	LO	189

*Note:* Semiquantitative determination of isovalerylglycine was performed using GC‐MS (12).

### 3.2 Dietary Data

**FIGURE 1:**
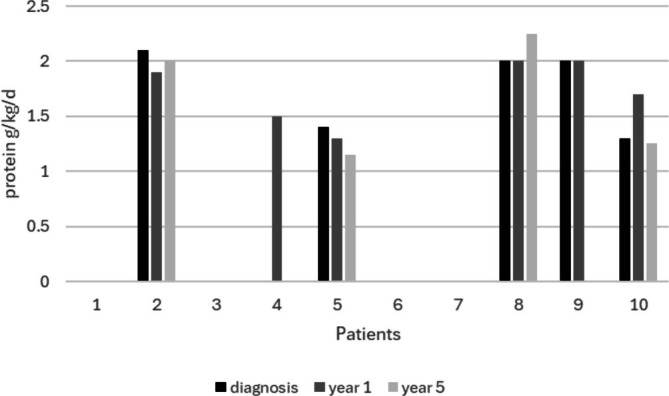
Protein restriction at diagnosis, 1 year and 5 years after diagnosis. Patients 1, 3, 4,6,7, 9 were not prescribed a protein restricted diet at diagnosis. Patients 4 and 9 had a protein restriction at 1 year, and Patients 1 and 4 are less than 5 years of age.

All patients were given instructions for sick day management including reduced protein and high energy intake regardless of NBS C5 level. An emergency plan was in place for all 10 patients. Three patients with a NBS C5 level less than 6 μmol/L had a moderate protein restriction (1.5–2.0 g/kg) 1 year after diagnosis, but this was relaxed by last review (Figure 1). Three patients with NBS C5 greater than 5.6 μmol/L (Patients 2, 5, 10) maintained a protein restriction ranging from 1.2 to 1.9 g/kg even at last review (Figure 1), predominantly due to a self‐restricted protein diet with some aversion noted.

### 3.3 Comparative Analysis of Phenotypes

A comparative analysis was performed between the “Classic” (*n* = 3) and “Mild” (*n* = 7) biochemical phenotypes (Table 2). The median initial C5 level was substantially higher in the Classic group (17.0 μmol/L; range 9.92–63.6) compared to the Mild group (3.7 μmol/L; range 0.959–4.39).

In terms of clinical stability, the Classic group had a higher median number of illness‐related admissions (Median: 9; range 2–10) compared to the Mild group (Median: 3; range 0–5) (Table 2). Furthermore, biochemical instability was restricted to the Classic group; 100% of the Mild group maintained normal ammonia levels (< 50 μmol/L) and no acidosis during all admissions. Despite these differences in clinical severity, there was no difference in neurodevelopmental outcomes between groups (Table 1), with 100% of patients across both phenotypes exhibiting “No Impairment” at last follow‐up.

### 3.4 Phenotypic Stratification and Clinical Stability During Intercurrent Illness

A clear distinction in clinical stability was observed when patients were stratified by their biochemical phenotype. The Classic IVA group (*n* = 3) exhibited a higher risk of metabolic instability, experiencing six documented episodes of hyperammonemia with peak ammonia levels ranging from 60 to 189 μmol/L. These episodes were predominantly triggered by viral gastroenteritis and febrile illnesses.

In contrast, the Mild IVA group (*n* = 7) remained biochemically stable. While this group required hospital admissions for similar triggers (including one instance of urticaria), none of these episodes resulted in hyperammonemia (ammonia levels < 50 μmol/L). Any mild acidosis observed in this cohort was transient and managed with precautionary intravenous fluids and carnitine.

Pharmacological requirements also mirrored this stratification: all Classic patients required dual therapy with l‐carnitine and glycine, whereas 43% (3/7) of the Mild cohort remained well without regular maintenance medication. Despite these differences in acute clinical course and treatment intensity, there was no difference in long‐term outcomes, with all patients being asymptomatic at their most recent follow‐up.

## 4 Discussion

This case series describes the clinical and neurological outcomes of 10 Australian patients with IVA, stratified by their C5 acylcarnitine levels identified on NBS. Our data highlight considerable heterogeneity in biochemical and clinical features, with minimal correlation between initial C5 levels and long‐term neurological outcomes. These findings are consistent with previous reports questioning the prognostic value of isolated NBS markers [6, 9].

Despite a wide range of C5 acylcarnitine levels on NBS (1.0–63.6 μmol/L), all patients in our cohort demonstrated normal neurological outcomes on follow‐up, including those with markedly elevated C5 values. Some individuals experienced significant metabolic instability, such as acidosis and hyperammonemia (peak ammonia: 189 μmol/L), yet none developed neurological abnormalities. These findings underscore the importance of early diagnosis through NBS and prompt metabolic management in preventing long‐term neurodevelopmental complications in IVA. They also suggest that current treatment protocols may be effective in maintaining neurological health, even in patients with considerable biochemical instability. The clinical trajectory of our cohort reinforces the value of risk stratification. While the three “Classic” patients presented symptomatically at the time of the initial NBS report, the seven “Mild” variants remained entirely asymptomatic throughout the diagnostic window. Despite these disparate beginnings, it is noteworthy that all 10 patients were entirely asymptomatic at their most recent clinical follow‐up. This universal achievement of long‐term clinical and neurological stability, even in those who presented with neonatal symptoms, highlights the success of the Australian NBS and metabolic management protocols.

Conversely, Patients 2 and 5 presented with marked elevations in C5 (9 and 17 μmol/L, respectively). While lower than the highest values in our cohort, these levels are substantially above screening thresholds and accurately predicted a classical IVA phenotype, characterized by repeated acidotic episodes, elevated anion gaps, and intermittent hyperammonemia. This demonstrates that initial NBS C5 concentrations can indeed serve as a valuable indicator of clinical risk, identifying infants who require intensive metabolic surveillance. Despite this biochemical and clinical instability, both patients maintained normal neurological outcomes, likely reflecting the efficacy of early identification and subsequent management of catabolic crisis [1, 4].

By stratifying our cohort into “Classic” and “Mild” phenotypes, we identified distinct clinical patterns that support a risk‐based management approach. Our analysis demonstrates a clear correlation between initial C5 levels and the frequency of true metabolic decompensations, while the “Classic” group accounted for all instances of hyperammonemia; the “Mild” group remained biochemically stable. Furthermore, the observation that nearly half of the “Mild” cohort required no maintenance medication, yet achieved identical “No Impairment” outcomes, confirms that C5 stratification can successfully identify a subset of patients for whom treatment intensity may be safely reduced. This analysis directly addresses our primary aim by providing a framework to differentiate those requiring intensive metabolic surveillance from those suitable for a more conservative, monitoring‐focused strategy.

Neurocognitive outcomes in symptomatic IVA have been shown to vary with disease severity and timing of diagnosis. Grunert et al. [17] reported that patients with neonatal onset IVA are at increased risk for deficits in executive function, processing speed, and working memory, particularly when metabolic control is suboptimal. In contrast, the implementation of NBS has led to the identification of individuals with milder biochemical phenotypes, often without clinical symptoms [2, 3].

Ensenauer et al. [9] described a common missense mutation (c.932C > T; p.Ala282Val) associated with a biochemically mild and potentially asymptomatic presentation. These findings have prompted a re‐evaluation of treatment intensity, as highlighted by Mütze et al. [6, 10], who demonstrated that genotype and diagnostic context predict neurological outcomes and can inform stratified management approaches. Their 2023 study proposed tailoring therapy based on biochemical and molecular markers to avoid overtreatment in low‐risk individuals while maintaining vigilance during catabolic stress. This evolving understanding of disease course underscores the importance of individualized care in IVA, balancing early intervention with phenotype‐specific management strategies [6].

The retrospective nature and small sample size of this study limit the generalizability of our findings. Additionally, neurological assessments were noted as “N” (normal) in medical records, but standardized neurodevelopmental testing was not uniformly applied. The reliance on surrogate clinical documentation may under‐detect subtle cognitive or behavioral impairments. Genetic data were not available for stratification, limiting phenotype–genotype correlation [6, 7].

## 5 Conclusions

This case series of 10 Australian patients with IVA detected through NBS highlights the disconnect between biochemical severity and clinical outcomes. Despite variability in C5 acylcarnitine levels and episodes of metabolic decompensation, all patients showed normal neurodevelopment, emphasizing the benefits of early diagnosis and management. These findings challenge the prognostic value of isolated C5 levels and support a more nuanced, individualized approach to IVA care. Although limited by the lack of genotype data and standardized assessments, the uniformly positive outcomes are reassuring. Future studies should include prospective designs and molecular analyzes to improve risk stratification and treatment strategies.

## Author Contributions

**Sharmila Kiss and Maureen Evans:** directly involved in the patient's care. **Sharmila Kiss and Maureen Evans:** planning of manuscript. **Sharmila Kiss, Gary Lazarovski, and Maureen Evans:** drafting of manuscript. **Sharmila Kiss, Gary Lazarovski, and Maureen Evans:** revision of the manuscript. All the authors also: approve the version to be published and agree to be accountable for all aspects of the work in ensuring that questions related to the accuracy or integrity of any part of the work are appropriately investigated and resolved.

## Funding

The authors have nothing to report.

## Ethics Statement

This project was formally classified as a Minimal Risk/Quality Improvement project by The Royal Children's Hospital Research Governance and Ethics Office.

## Consent

All procedures were followed in accordance with the ethical standards of the responsible committee on human experimentations (institutional and national) and with the Helsinki Declaration of 1975, as revised in 2000. Informed consent was obtained from the patients for being included in the article.

## Conflicts of Interest

The authors declare no conflicts of interest.

## Data Availability

The data that support the findings of this study are available from the corresponding author upon reasonable request.
